# Using RSM for Optimum of Optimum Production of Peptides from Edible Bird’s Nest By-Product and Characterization of Its Antioxidant’s Properties

**DOI:** 10.3390/foods11060859

**Published:** 2022-03-18

**Authors:** Jie Cao, Ning Xiong, Yu Zhang, Yuwei Dai, Yuye Wang, Lingyu Lu, Lin Jiang

**Affiliations:** 1 School of Pharmaceutical Sciences, Sun Yat-Sen University, Guangzhou 510006, China; caoj35@mail2.sysu.edu.cn (J.C.); zhangy999@mail2.sysu.edu.cn (Y.Z.); daiyw6@mail2.sysu.edu.cn (Y.D.); wangyy253@mail2.sysu.edu.cn (Y.W.); luly6012@163.com (L.L.); 2 School of Public Health, Southern Medical University, Guangzhou 510515, China; 3190090032@i.smu.edu.cn

**Keywords:** edible bird’s nest by-products, response surface methodology, antioxidant peptides, radical scavenging ability, PC12 cells

## Abstract

In this research, the neutrase hydrolysis conditions of edible bird’s nest (EBN) by-products were optimized by response surface methodology (RSM). Antioxidant peptides were then isolated from the EBN by-products by ultrafiltration and chromatography taking the DPPH radical scavenging ability as an indicator. The antioxidant activity of the purified peptides was estimated by radical scavenging ability and sodium nitroprusside (SNP)-induced damage model in PC12 cells. When the enzyme concentration was10 kU/g-hydrolysis temperature was 45 °C, and hydrolysis time was 10.30 h, the degree of hydrolysis (DH) of EBN by-product hydrolysate (EBNH) was the highest. The purified peptide exerted strong scavenging ability with EC_50_ values of 0.51, 1.31, and 0.65 mg/mL for DDPH, ABTS, and O_2_^−^ radicals, respectively. In addition, the purified peptides could significantly reduce the SNP-induced oxidative damage of PC12 cells, and twelve peptides that were rich in leucine (Leu), valine (Val), and lysine (Lys) were identified by LC-MS/MS. These results suggested that EBN by-products have potential as new materials for natural antioxidant peptides.

## 1. Introduction

Excessive reactive oxygen species (ROS) and reactive nitrogen species (RNS) have been implicated in the oxidative damage of cells, the occurrence of inflammation, as well as the pathophysiology of endothelial barrier dysfunction [1], which may further contribute to the development of cancer, atherosclerosis, Alzheimer’s disease Heimer’s disease, etc., [2,3]. Accumulating evidence demonstrated that moderate intake of substances with antioxidant activity can effectively neutralize the extra free radicals in the body, attenuate lipid peroxidation, and prevent the occurrence of diseases [4]. Although the synthetic antioxidants, including butylated hydroxyanisole (BHA), butylated hydroxytoluene (BHT), and tert-Butylhydroquinone (TBHQ), possessed the advantages of high efficiency and low price, the potential carcinogenicity server limited its clinical and daily usage [5,6]. Therefore, the search for high-efficiency and low-toxic natural antioxidants has captured more and more attention. Antioxidant peptides obtained by enzymatically hydrolyzing proteins showed promising pharmacokinetic and pharmacodynamic properties, such as being easily absorbed by our body, promising anti-oxidative activity, and few side effects, and was demonstrated as a substitute for synthesizing antioxidants [7,8,9]. Up to now, many antioxidant peptides have been separated and identified from different animal, plant, and fungal proteins including rice bran [10], seafood and their processing by-products [11,12], and mushrooms [13].

EBN is composed of the saliva of some species including *Aerodramus* and *Collocalia* during reproduction, which is mixed with impurities like feathers and dirt [14]. EBN contains protein, sialic acid, minerals, and other nutrients [15]. It is reported that EBN may benefit human health by its anti-oxidative [16], anti-viral [17], anti-aging [18], and cell-proliferation-promoting activities [19]. Recently, the antioxidant activity of EBN has attracted research attention and some progress has been made. For example, EBN extracts were demonstrated to alleviate high-fat diet-induced oxidative stress and inflammation by regulating the transcription of some hepatic antioxidant and inflammation-related genes [20]. In addition, Albishtue et al. confirmed that EBN could increase the concentration of plasma antioxidants in non-pregnant rats and reduce the level of oxidative stress [21]. However, most of these studies are based on the overall active compositions of EBN, and the peptides with antioxidant activity in EBN are rarely reported in existing research.

EBN produces a great deal of EBN by-products during the processing, which is difficult to be effectively utilized or discarded, resulting in a certain amount of waste [22]. Studies have shown that there is no significant difference in the nutritional composition of EBN by-products and EBN. Because the EBN by-products have high protein content (50%), they can act as a precursor to antioxidant peptides [23]. Based on the research status of EBN peptides and natural antioxidant peptides, to further confirm that peptides are the active components of EBN antioxidants and effectively develop a new type of natural antioxidant—EBN peptide, our study used EBN by-products as raw materials to optimize the enzymatic hydrolysis process of EBN by-product peptides. Moreover, the best antioxidant active components were screened, which antioxidant activity was estimated by in vitro method, and sequences were identified by LC-MS/MS. This research can not only reduce the waste of EBN by-products but also provide data support for the further processing of EBN by-products. 

## 2. Materials and Methods 

### 2.1. Materials

Indonesia’s 006 factories provided EBN by-products, which were identified as genuine by Lin Jiang, an associate professor of Sun Yat-Sen University. Six proteases (Papain, acid protease, neutrase, alcalase, pepsin, and trypsin), superoxide anion kits, and Sephadex G-25 were obtained from Beijing Solarbio Science & Technology Co., Ltd. (Beijing, China). 2,2-diphenyl-1-picrylhydrazyl (DPPH), O-phthaldialdehyde (OPA), ascorbic acid (VIT C), and HPLC-grade trifluoroacetic acid (TFA) were acquired from Macklin (Shanghai, China). 2,2′-azino-bis (3-ethylbenzthiazoline-6-sulfonic acid (ABTS) kits were obtained from Beyotime Bio-Technology Co., Ltd. (Shanghai, China). Serine standard was acquired from Yuanye Biotechnology Co., Ltd. (Shanghai, China). HPLC-grade acetonitrile was obtained from Oceanpak (Stockholm, Sweden). Edaravone was acquired from Aladdin (Shanghai, China). Sodium nitroprusside (SNP) was acquired from Sigma-Aldrich Co. (St. Louis, MO, USA). Other reagents and solvents were of analytical grades. 

### 2.2. Preparation of EBNH

In this study, six proteases: papain, acid protease, neutrase, alcalase, pepsin, and trypsin were used to screen the optimal one. About 1 g of dried EBN by-product powder was mixed with 50 mL of deionized water and refrigerated at 4 °C for 24 h. Then the mixture was heated at 100 °C for 30 min. When the mixture was chilled down to room temperature, 1 mol/L hydrochloric acid or sodium hydroxide solution was added to regulate the pH value, and 8 kU/g of protease was added to hydrolyze for 10 h at a suitable temperature (Table 1). Enzymes were inactivated at 100 °C for 10 min and then centrifuged at 5000 r/min for 10 min [24]. Supernatants were collected and lyophilized to measure DPPH radical scavenging activity and DH.

### 2.3. Single-Factor Experiments

Neutrase was selected as the optimal protease in this part, and the water/material ratio, enzyme concentration, hydrolysis temperature, hydrolysis time, and pH were chosen for single-factor experiments. The hydrolysis condition of neutrase was as follows: water/material ratio of 50.0, enzyme concentration of kU/g, hydrolysis temperature of 40 °C, hydrolysis time of 6.0 h, and pH of 7.0. The ranges of variables were as follows: water/material ratios of 20.0, 30.0, 40.0, 50.0, and 60.0, enzyme concentrations of 4, 6, 8, 10, and 12 kU/g; hydrolysis temperatures of 30, 40, 50, 60, and 70 °C; hydrolysis times of 2.0, 4.0, 6.0, 8.0, 10.0, and 12.0 h; pH of 5.0, 6.0, 7.0, 8.0, and 9.0. Exploring the best conditions for one variable requires fixing the other variables, and the specific hydrolysis way as described in Section 2.2.

### 2.4. Optimization Preparation Conditions by Box-Behnken Design

Based on the results of single-factor experiments, the fixed water/material ratio and the pH were set to 30 and 7.0, respectively. The three independent variables of the three-level Box Behnken design (BBD) were enzyme concentration, hydrolysis temperature, and hydrolysis time. Encode variables according to Equation (1).

(1) $$x_{i}=\frac{X_{i}-X_{0}}{△X_{i}}$$

where *x_i_*, *X_i_*, *X*_0_ and Δ*X_i_* represented the coded value of a given variable, the actual value of the variable, the actual value of the center point *X_i_*, and the step change value, respectively. The range and level of independent variables are shown in Table 2, a total of seventeen experimental points were tested in random order.

Data of BBD were analyzed by multiple regressions to fit the following quadratic polynomial Equation (2).

(2) $$Y=\beta_{0}+\sum_{i=1}^{k}\beta_{i}X_{i}+\sum_{i=1}^{k}\beta_{ii}X_{i}{}^{2}+\sum_{i=1}^{K-1}\sum_{j>i}^{k}\beta_{ij}X_{i}X_{j}$$

*Y*: response function; β_0_: intercept. *X_i_/X_j_*: the coded independent variables. where β*_i_*, β*_ii_*, and β*_ij_* indicated the coefficients of the linear term, the quadratic term, and the interaction term, respectively. The relationship between the response of each factor and the experimental level was reflected by the response surface and contour plots of the fitted polynomial equations, and the optimal conditions are derived from the equations. The regression coefficients of individual linear, quadratic, and interaction terms were performed by analysis of variance.

### 2.5. Degree of Hydrolysis

A slight modification of the previous method was used for the determination of DH [25]. OPA reagent consisted of two solutions A and B. Solution A was 1.905 g of dissolved sodium tetrahydroborate and 50 mg of sodium dodecyl sulfate (SDS) in 30 mL deionized water; solution B was 40 mg of dissolved OPA in 1 mL ethanol absolute. Then, after mixing solutions A and B, 44 mg β-mercaptoethanol was added and the solution volume was made to 50 mL with deionized water to obtain the OPA reagent working solution [26]. OPA reagent (150 μL) was added to the sample solution (20 μL) at a concentration of 1 mg/mL, followed by incubation at room temperature and measuring the absorbance at 340 nm using a microplate reader. Serine was used as the standard control and deionized water as the blank control. The formula for calculating DH is as follows:

h = ((A_1_ − A_0_/A_2_ − A_0_) × 0.91516 − β)/α (3)

DH (%) = h/h_tot_ × 100 (4)

where A_0_, A_1_, and A_2_ are the absorbance of blank control, sample, and standard control, respectively. The α, β, and h_tot_ for EBN by-products are 0.4, 1.0, and 7.6, respectively.

### 2.6. DPPH Radical Scavenging Assay

Determination of DPPH radical scavenging activity was in line with Zhang et al. [27]. Briefly, a solution of 0.1 mM DPPH in ethanol absolute was freshly prepared. After mixing DPPH solution (100 µL) and sample solution (100 µL), the absorbance of the mixture was measured at 517 nm after 30 min’s incubation in the dark. VIT C was used as a positive control, while deionized water was used as vehicle control. The scavenging activity of DPPH radical was calculated as follows:

DPPH radical scavenging activity (%) = 1− [(A_3_ − A_2_)/(A_1_ − A_0_)] × 100% (5)

A_0_: the absorbance of deionized water and ethanol absolute; A_1_: the absorbance of DPPH solution and deionized water; A_2_: the absorbance of ethanol absolute and sample solution; A_3_: the absorbance of DPPH solution and sample solution.

### 2.7. ABTS Radical Scavenging Assay

Determination of ABTS radical scavenging activity was based on the kit instructions. Briefly, a fresh ABTS working solution (200 µL) and sample solution (10 µL) were mixed. After incubation in dark for 10 min, the absorbance at 734 nm was measured. VIT C was a positive control. The scavenging activity of ABTS radical was calculated as follows:

ABTS radical scavenging activity (%) = 1 − [(A_2_ − A_1_)/A_0_] × 100% (6)

where A_0_, A_1_, and A_2_ represented the absorbance of deionized water and ABTS working solution, deionized water and sample solution, and sample solution and ABTS working solution, respectively.

### 2.8. O_2_^−^ Radical Scavenging Assay

Determination of O_2_^−^ radical scavenging activity according to the manufacturer’s instructions. About 40 µL tetramethyl ethylenediamine (TEMED) solution, 10 µL ammonium persulfate (AP) solution, 50 µL hydroxylamine hydrochloride were mixed to form 25 µL sample solution and incubated for 30 min at 37 °C. After adding 50 µL sulfonic acid and α-naphthylamine, the mixture was incubated at 37 °C for another 20 min. Then the absorbance was determined at 530 nm. VIT C was a positive control. The scavenging activity of O_2_^−^ radical was calculated as follows:

O_2_^−^ radical scavenging activity (%) = 1 − (A_0_/A_1_) × 100% (7)

where A_0_ and A_1_ are the absorbance of the control and sample group, respectively. Deionized water replaced the sample solution of the control group.

### 2.9. Peptides Separation by Membrane Ultrafiltration

EBNH was separated into two fractions (>10 kDa (EBNH-1) and <10 kDa (EBNH-2)) by a 10 kDa ultrafiltration membrane (Millipore, Merck, Germany) at 4500× *g* 30 min. DPPH radical scavenging activity was determined after the two components were freeze-dried, and the component with stronger activity was chosen for purification in the next step.

### 2.10. Purification by Gel Filtration Chromatography

EBNH-2 (100 mg/mL) was filtered and eluted at a flow rate of 1.5 mL/min on a Sephadex G-25 gel column (2.6 × 60 cm) with distilled water as a mobile phase. A tube of eluate was collected (3 mL) every two minutes and its absorbance were measured at 280 nm. Five fractions (F1–F5) were freeze-dried to evaluate their DPPH radical scavenging activity.

### 2.11. Purification by RP-HPLC

The filtrate (50 μL) of the F3 fraction (100 mg/mL) was separated and purified on an LC-20AT HPLC system using an ultimate LP-C18 column (10 × 250 mm, 5 µm). The liquid phase conditions are as follows: (1) flow rate: 1.5 mL/min; (2) mobile phase A: 0.1% TFA in water; mobile phase B: 0.1% TFA in acetonitrile; (3) elution program: 3–25% solvent B, 55 min; 25–3% solvent B, 5 min. The eluate was collected to measure its absorbance at 280 nm, and DPPH radical scavenging activity was performed after lyophilization.

### 2.12. Cell Culture and Cytotoxicity Assay

PC12 cells were obtained from the Cell Resource Center of Shanghai Institute of Biological Science, Chinese Academy of Science, and maintained according to their instructions. Cell viability was monitored with cell counting kit-8 (CCK-8) (40203ES80, Yepsen Biotech Co., Ltd., Shanghai, China). In brief, PC12 cells were cultured in a DMEM medium containing 5% FBS, 5% HS, and 1% penicillin and streptomycin at 37 °C in a moist environment with 5% CO_2_. After being seeded in 96-well plates (5 × 10^3^ cells/well) for 24 h, the old medium was replaced by a medium containing indicated concentrations of the P2 fraction (50~400 μM) for another 24 h. Then CCK-8 solution (10 μL) was added to the culture medium and continued to incubate at 37 °C for 1 h. Finally, the OD value was determined at 450 nm. The formula for calculating cell viability is as follows:

Cell viability = (A_1_/A_0_) × 100% (8)

where A_0_ and A_1_ are the absorbance of untreated cells and peptide-treated cells, respectively.

### 2.13. Cytoprotective Effects against PC12 Cells Damage Induced by SNP

PC12 cells were seeded in 96-well microplates (5 × 10^3^ cells/well) and cultured for 24 h. The old medium was removed and PC12 cells were stimulated with SNP at the concentrations of 100~800 μM for another 24 h. Cell viability was monitored and the concentration with cell viability close to 60% was selected as the suitable concentration of SNP [28].

The oxidative damage of PC12 cells was induced at the optimal SNP concentration to evaluate the protective effect of the P2 fraction. The PC12 cells were grown in 96-well plates (5 × 10^3^ cells/well) for 24 h and then treated with purified peptides (50~400 μM) for 2 h. After adding the SNP to the medium and continuing to the culture at 24 h, the viability of cells was determined with a CCK-8 kit.

### 2.14. Peptide Sequence Identification by LC-MS/MS

The P2 fraction with the most anti-oxidative activity was loaded on an Acclaim Pep Map C18 column (3 µm, 100 A, 75 µm × 150 mm) and analyzed on an Ultimate 3000 LC system using a mass spectrometer (Thermo Scientific Q Exactive, Waltham, MA, USA). Analysis conditions were as follows: (1) flow rate: 300 nL/min; (2) eluent A: 0.1% formic acid water; eluent B: acetonitrile/water/formic acid solution with a volume ratio of 80:20:0.1; (3) elution program: 5–45–50 min, 5–50–90% eluent B; 55–65 min, 90–5% eluent B; (4) spectral recording range: 100–2000 (*m*/*z*). Before MS/MS analysis, the MS scanning range (M/Z 350–2000) was determined by high-resolution (60,000) Q-Exactive. Peptide sequence and molecular weight were determined according to MASCOT search engine software and uniport databases.

### 2.15. Statistical Analysis

The pooled data were indicated as means ± standard deviation (SD) from at least three experiments. Multigroup analyses were performed with one-way analysis of variance (ANOVA) with Tukey posthoc test in SPSS software (version 22.0, SPSS Inc., Chicago, IL, USA). Statistical difference was regeared as the *p* < 0.05.

## 3. Results and Discussion

### 3.1. Selection of Proteolytic Enzymes

**Figure 1:**
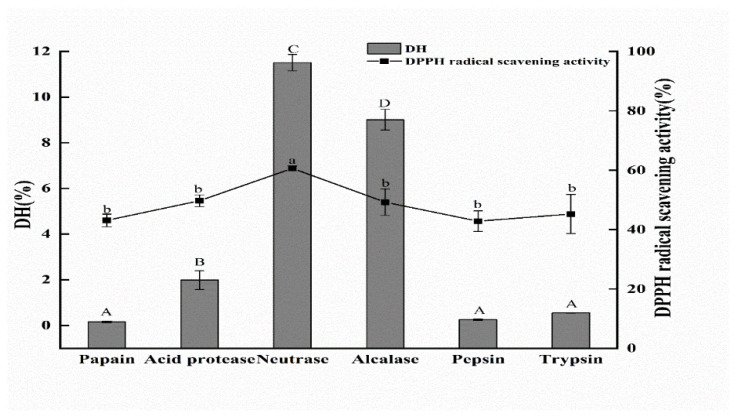
DH and DPPH radical scavenging activities of six protease hydrolysates. DH represented the degree of hydrolysis; DPPH radical scavenging activity assay was measured at concertation of hydrolysates was 3 mg/mL. The data indicated as means ± SD (*n* = 3). The column values of ^a–b^ or ^A−D^ with the same superscript indicated an insignificant difference (*p* > 0.05).

The selection of a suitable protease is very important because the DH and antioxidant activity of protein hydrolysates are associated with the type of protease [29]. Six different proteases (papain, acid protease, neutrase, alcalase, pepsin, and trypsin) were applied to hydrolyze EBN by-products in this research. It can be seen from Figure 1 that DH of neutrase hydrolysate was the highest at 11.51 ± 0.36%, which was observably higher than other hydrolysates (*p* < 0.05). Furthermore, the hydrolysates of six different proteases exhibited different DPPH radical scavenging activities. The order of activities for six hydrolysates to scavenge DPPH radical was neutrase > alcalase > acid protease > trypsin > papain > pepsin, which agreed with the result that the hydrolysate with a high DH exhibits higher antioxidant activity [30]. Thus, neutrase was selected for subsequent experiments in further study.

### 3.2. Single-Factor Experiments

**Figure 2:**
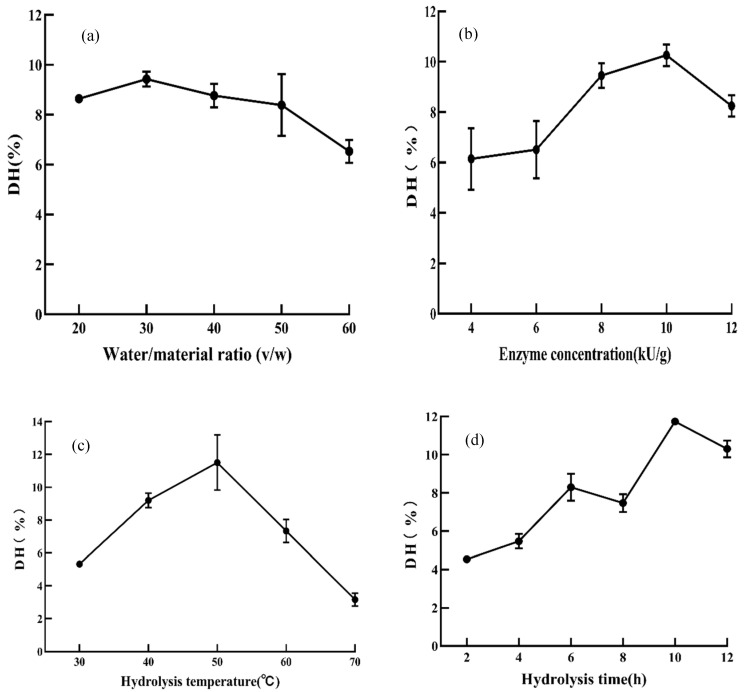

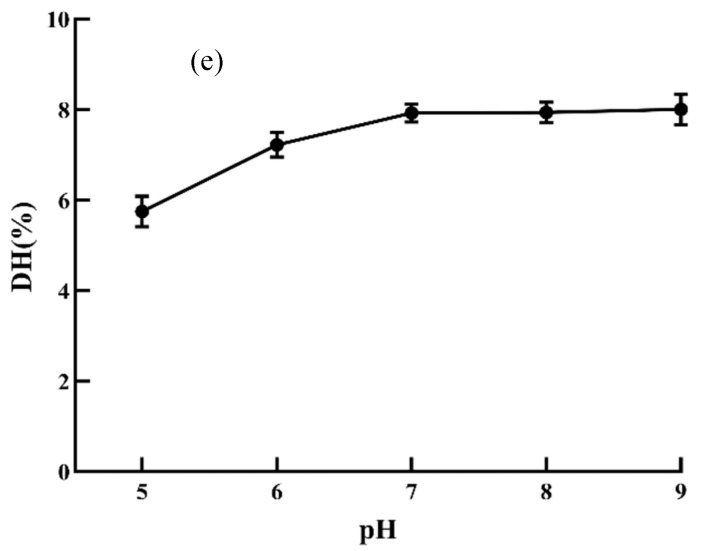
Effects of five single factors on DH of EBNH. (**a**) Water/material ratio (*v/w*); (**b**) enzyme concentration (U/g); (**c**) hydrolysis temperature (°C); (**d**) hydrolysis time (h); (**e**) pH; DH represents the degree of hydrolysis. Data indicated as means ± SD (*n* = 3).

Optimizing the enzymatic hydrolysis process is crucial to obtain desired peptides. It can be seen from Figure 2a that DH raised with the increase of water/material ratio. When the water/material ratio was 30.0, DH reached the highest 9.43 ± 0.30%. While the water/material ratio exceeded 30.0, the substrate concentration was too low, which reduced the collision probability between protease and substrate, partially inhibited the enzymatic hydrolysis reaction, and reduced the DH [31]. Therefore, the optimum water/material ratio was 30.0.

As displayed in Figure 2b, DH was raised from 6.14 ± 1.23% to 10.26 ± 0.44% while the enzyme concentration was added from 8 kU/g to 10 kU/g, which may be due to the sufficient binding of EBN by-products proteins and proteases to complete the hydrolysis. With the continuous increase of protease, the reaction substrate had been fully hydrolyzed, and the generated peptides were further hydrolyzed into amino acids, resulting in a decrease in DH [32]. Therefore, the optimum enzyme concentration was 10,000 U/g.

As shown in Figure 2c, with the increase of hydrolysis temperature, DH showed a trend of first increasing and then decreasing. DH was up to 11.50% at 50 °C. Above 50 °C, the high temperature denatured and inactivated the enzyme, resulting in a decrease in the DH. Thus, the optimum temperature was 50 °C.

As displayed in Figure 2d, DH showed a trend of first increasing and then decreasing with the prolongation of hydrolysis time. When the hydrolysis time was 10 h, the highest DH was 11.33 ± 0.08%. Peptides were re-digested with an increased hydrolysis time, leading to a decrease in DH [32]. In addition, the hydrolysate competed with the substrate for the active site of the enzyme, inhibiting the enzymatic hydrolysis reaction [33] and reducing DH. Therefore, the optimum hydrolysis time was 10 h.

It can be seen from Figure 2e that DH was affected by pH insignificantly. The growth of DH was slow with the increase of pH, which might be related to the fact that the medium and high concentration of alkali could destroy the structure of protein and produce new peptides [34]. Considering that the optimum pH range of neutrase was 6~7.5, therefore, the optimum pH was 7.

### 3.3. Optimization of Hydrolysis Parameters by RSM

Optimal experimental parameters for enzymatic hydrolysis were analyzed by RSM, and the BBD results are shown in Table 3. DH ranged from 10.60 ± 0.17% to 17.35 ± 0.13% under different conditions. The function of DH (Y, %) with enzyme concentration (U/g, A), hydrolysis temperature (°C, B), hydrolysis time (h, C) was established based on the results of multiple regression analysis. The formula was as follows:

Y = 17.08 + 0.46A − 2.26B + 0.05C + 0.51AB − 0.34AC − 0.36BC − 0.8A^2^ − 2.68B^2^ − 0.53C^2^ (9)

The results of the analysis of variance were displayed in Table 4. With an increase of *F* value and a decrease of *p*-value, the corresponding variables become more significant [35]. According to Table 4, the quadratic regression model *F* = 51.21 and *p* < 0.0001, suggesting that the model was significant and fitted well with the second-order equation. Furthermore, *R*^2^ = 0.9850; *R*^2^_adj_ = 0.9685, indicating that the predicted value had a good correlation with the actual value, and the results were true and reliable. The effects of A, B, AB, A^2^, B^2^, and C^2^ on the DH were significant if (*p* < 0.05), else the effects were insignificant (*p* > 0.05). According to the *F* value, the order of influence of various factors on DH was hydrolysis temperature > enzyme concentration > hydrolysis time.

**Figure 3:**
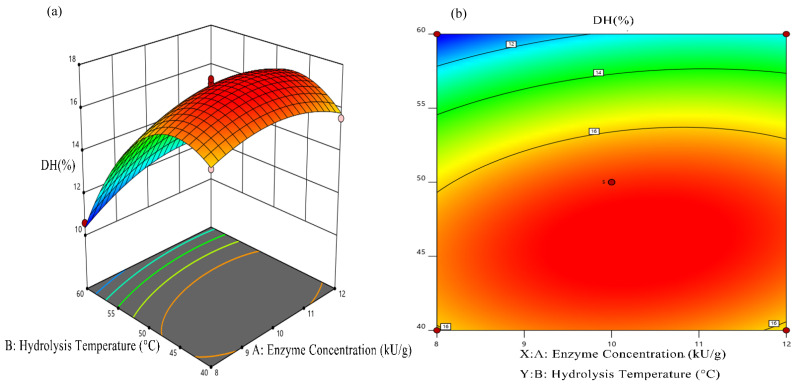

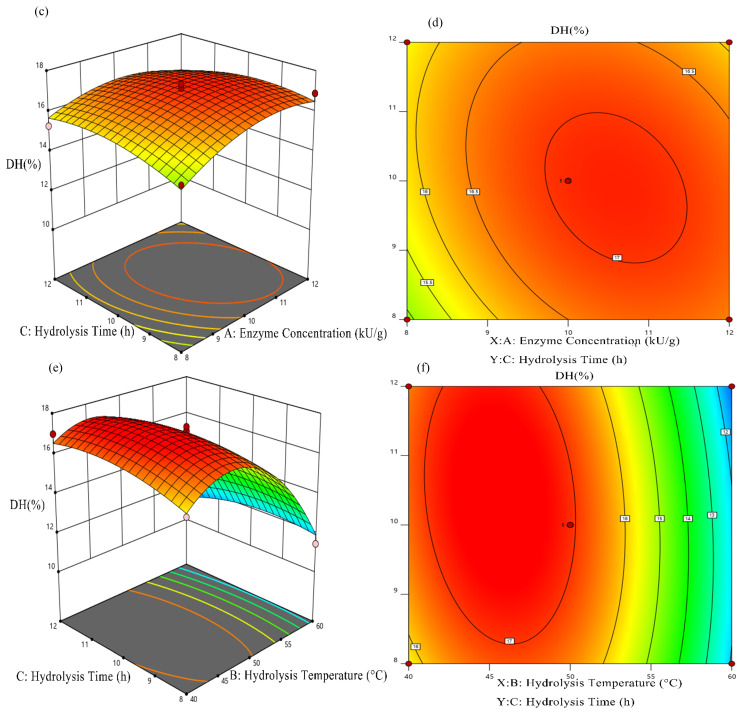
Responsive surface plots and corresponding contour plots of DH of BENH. Interaction of enzyme concentration and hydrolysis temperature (**a**,**b**). Interaction of enzyme concentration and hydrolysis time (**c**,**d**). Interaction of hydrolysis temperature and hydrolysis time (**e**,**f**).

Interaction among various factors of the regression model was revealed by the steepness of the response surface and the shape of the contour line. The steeper the response surface, the closer the contour to the ellipse, and the more significant the interaction between variables. The interaction between hydrolysis temperature and enzyme concentration exerted the most influence on the hydrolysis, shown as a steep surface and an oval contour in the diagram (Figure 3a,b). However, the interaction between hydrolysis temperature and hydrolysis time exhibited little effect on the hydrolysis, shown as a flat surface and circular contour in the diagram (Figure 3c,d). The effect on the hydrolysis exerted by the interaction between enzyme concentration and hydrolysis time was moderate, shown as a less steep surface and a less circular contour in the diagram (Figure 3e,f), which agreed with the variance analysis.

The optimal hydrolysis conditions of EBNH were determined by Design Expert-12.0 software as follows: enzyme concentration was 10.24 kU/g, hydrolysis temperature was 45.80 °C, hydrolysis time was 10.30 h. under these conditions, the predicted value of DH was 17.58%. Regarding the feasibility of this experiment, the optimal process parameters were adjusted as the enzyme concentration was 10 kU/g, hydrolysis temperature was 45 °C, hydrolysis time was 10.30 h, and three verification experiments were implemented. The average content of DH was 17.37 ± 0.07%, which was near to the predicted value, suggesting that the prediction model could well reflect the actual situation.

### 3.4. Ultrafiltration of EBNH

**Figure 4:**
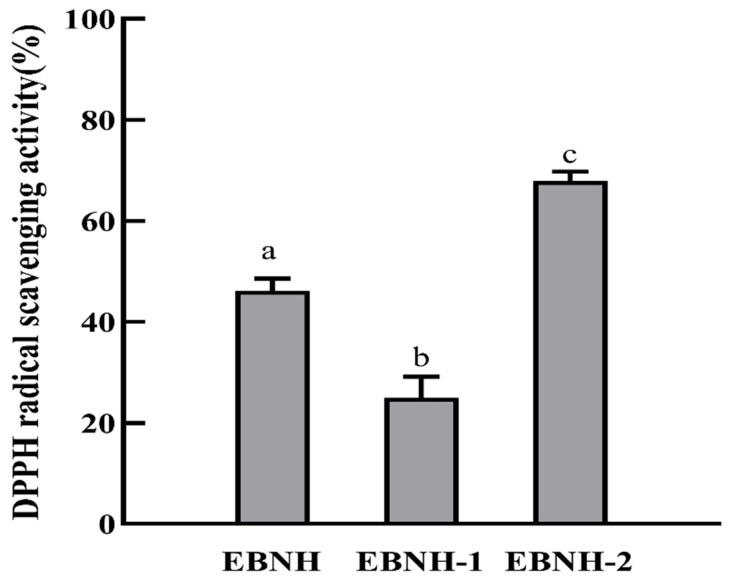
DPPH radical scavenging activities of EBNH, and its ultrafiltration fractions EBNH-1 and EBNH-2 at a concentration of 2 mg/mL. The data indicated as means ± SD (*n* = 3). The column values of a–c with the same superscript indicated an insignificant difference (*p* > 0.05).

In this study, EBNH was separated into two components (>10 kDa (EBNH-1) and <10 kDa (EBNH-2)) by a 10 kDa ultrafiltration membrane. As displayed in Figure 4, the DPPH radical scavenging activities of EBNH, EBNH-1, and EBNH-2 at the concertation of 2 mg/mL were 46.14 ± 2.42%, 24.97 ± 4.21%, and 67.93 ± 1.88%, respectively. Among them, the DPPH radical scavenging activities for EBNH and EBNH-1 fractions were markedly lower than those of the EBNH-2 fraction (*p* < 0.05). The result showed that high molecular weight components exhibited weaker antioxidant activity than low molecular weight components, which corresponded with the antioxidant activity of protein hydrolysate being inversely proportional to its average molecular weight distribution [36]. Therefore, the EBNH-2 fraction was selected for further purification.

### 3.5. Gel Filtration Chromatography of EBNH-2 Fraction

**Figure 5:**
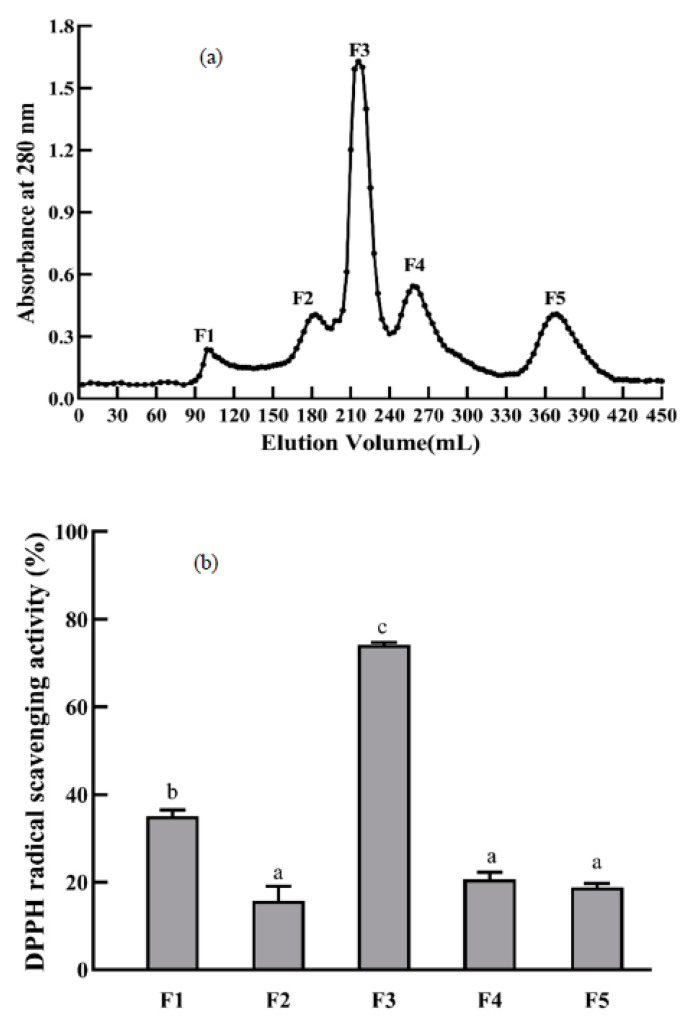
Elution profile of EBNH-2 on an Sephadex G-25 column (**a**); DPPH radical scavenging activities for EBNH-2 subfractions (F1–F5) at a concentration of 1 mg/mL (**b**). The data indicated as means ± SD (*n* = 3). The column values of a–c with the same superscript indicated an insignificant difference (*p* > 0.05).

Gel filtration chromatography is a common technique applied to separating substances from different molecular sizes, which has been extensively applied in the separation and purification of peptides and proteins due to its high selectivity and high resolution. It can be seen from Figure 5a that EBNH-2 was fractionated into five subfractions (F1–F5) by the Sephadex G-25 column. As displayed in Figure 5b, other subfractions of DPPH radical scavenging activities at the concretion of 1 mg/mL were significantly lower than the F3 fraction (*p* < 0.05). Thus, the F3 fraction was collected and further separated in an RP-HPLC column.

### 3.6. Separation of the F3 Fraction by RP-HPLC

**Figure 6:**
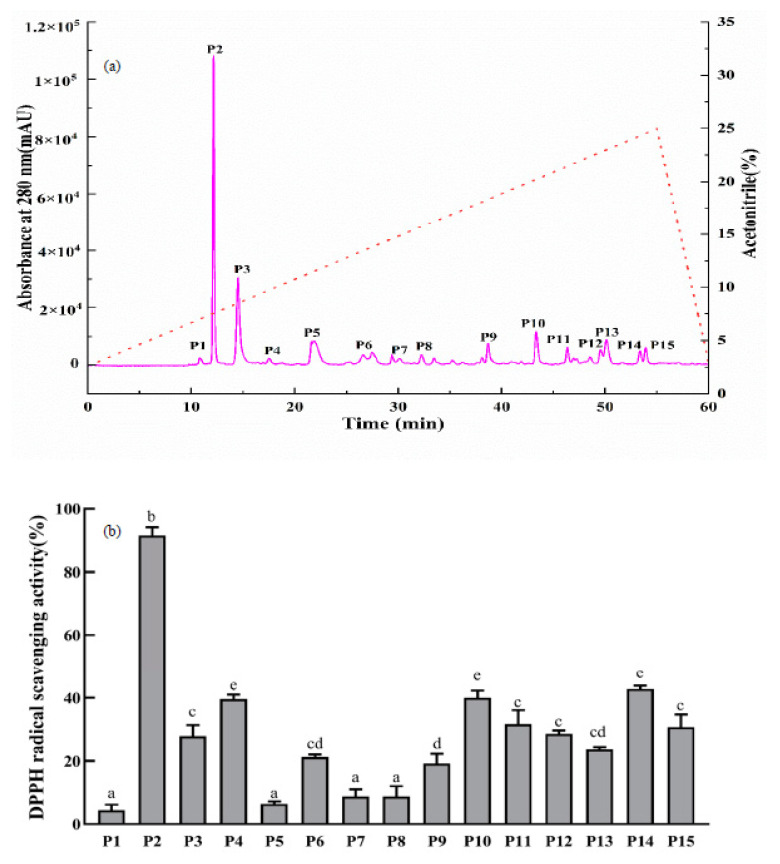
Elution profile of the F3 fraction separated by RP-HPLC (**a**); DPPH radical scavenging activities of the F3 subfractions (P1–P15) at a concentration of 1 mg /mL (**b**). The data indicated as means ± SD (*n* = 3). The column values of a–e with the same superscript indicated an insignificant difference (*p* > 0.05).

RP-HPLC was applied for separation and purification according to the hydrophobicity of peptides, and it is extensively used in the last purification step because of its virtues of ease of operation, high resolution, and high sensitivity. As displayed in Figure 6a, fifteen components (P1–P15) were obtained according to chromatographic peaks. As displayed in Figure 6a, the P2 fraction showed the strongest DPPH radical scavenging activity (91.60 ± 2.57%) when the concentration was 1 mg/mL in all fractions. Therefore, the P2 fraction was collected and lyophilized for further research.

### 3.7. Radical Scavenging Activity of the P2 Fraction

**Figure 7:**
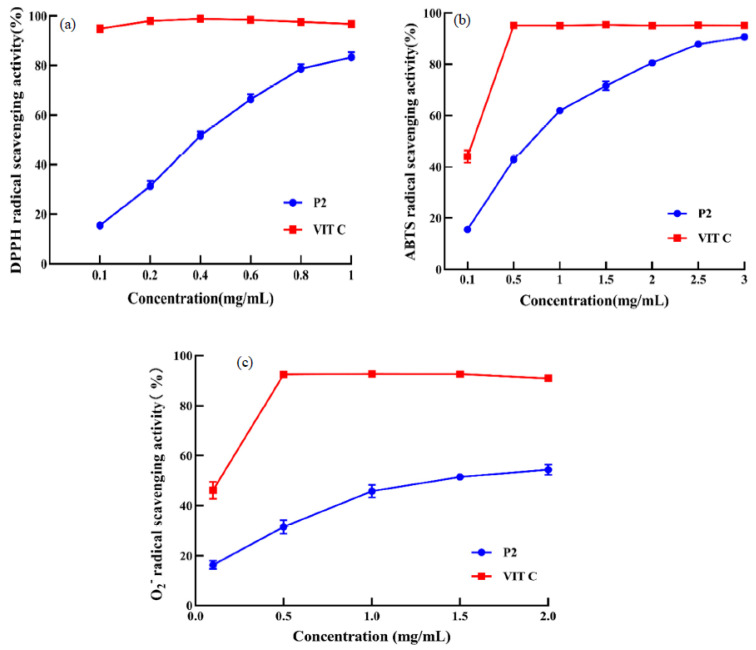
The DPPH (**a**), ABTS (**b**), and O_2_^−^ (**c**) radical scavenging activities of the P2 fraction from EBN by-products hydrolysates. The data indicated as means ± SD (*n* = 3).

A lot of methods are currently available to evaluate antioxidant activity. In this study, the antioxidant activity of the P2 fraction was preliminarily examined by three in vitro assays, namely DPPH radical, ABTS radical, and O_2_^−^ radical scavenging ability. As shown in Figure 7, three radical scavenging activities were found to increase with increasing the concentrations of the P2 fraction, while their radical scavenging activities were still lower than the same concentration of VIT C. The half-elimination ratio (EC_50_) value of the P2 fraction for DPPH radical scavenging was 0.51 mg/mL. The P2 fraction showed the highest scavenging activity against DPPH radicals at a concentration of 1 mg/mL, which reached at 83.30 ± 2.12% and close to the VIT C scavenging activity. The EC_50_ value of the P2 fraction for the DPPH radical scavenging was much lower than those peptides identified from red stingray cartilage, such as the EC_50_ of VPR and IEPH were 4.61 mg/mL and 1.90 mg/mL respectively [37], and it is also lower than that of PSYV (17.0 mg/mL) isolated from protein hydrolysates for loach [38]. Moreover, the EC_50_ value of the P2 fraction of the ABTS radical scavenging rate was 1.31 mg/mL, which was superior to that of EC_50_ for the peptides identified from protein hydrolysates of bluefin leatherjacket heads [39], loach meat [40], and phaseolus vulragis [41]. In addition, the EC_50_ value of the P2 fraction for O_2_^−^ radical scavenging ability was 0.65 mg/mL, which was superior to peptides from Spanish mackerel skin such as the EC_50_ of PFGPD and PYGAKG were 0.91 mg/mL and 0.80 mg/mL, respectively [42]. Meanwhile, the EC50 value was much lower than YLPYA (3.61 mg/mL), which was isolated from miiuy croaker swim bladders [43]. The results showed that the P2 fraction exhibited a distinguished ability to scavenge radicals.

### 3.8. In Vitro Cytotoxicity of the P2 Fraction and Protective Effect on PC12 Cells

**Figure 8:**
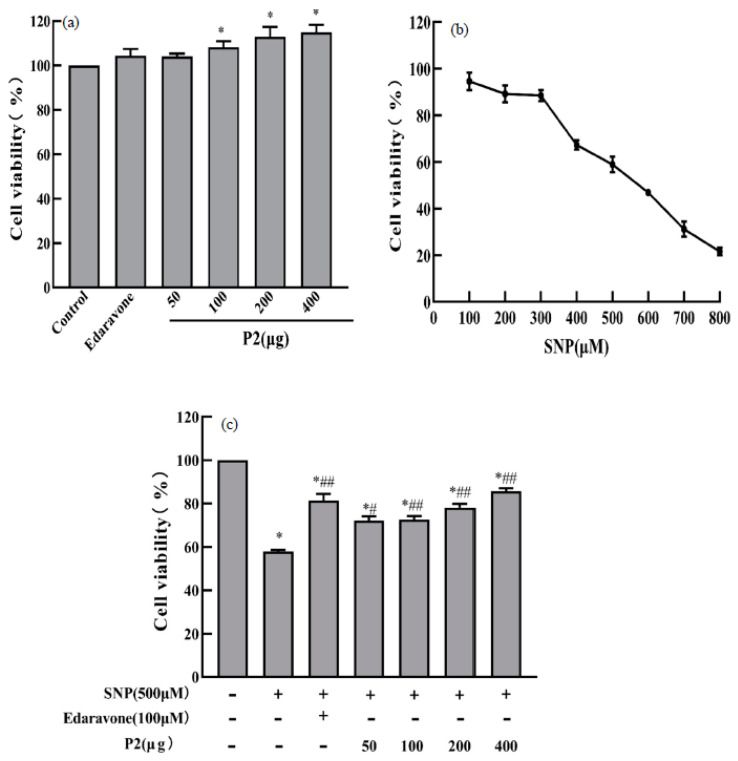
Cytotoxicity of P2 fraction from hydrolysate of EBN by-products in PC12 cells (**a**); damage effect of SNP on PC12 cells (**b**); protective effects for the P2 fraction against SNP-induced oxidative damage on PC12 cells (**c**). The data indicated as means ± SD (*n* = 3). * *p* < 0.05 vs. the control group. # *p* < 0.05 vs. the SNP stimulated group. ## *p* < 0.01 vs. the SNP stimulated group.

To further investigate the antioxidant effects of EBN by-product protein hydrolysate, the in vitro cytotoxicity, and protective effects on damaged cells of purified P2 fractions were assessed by CCK-8 assay. It can be seen from Figure 8a that the P2 fraction had no cytotoxic effect on PC12 cells even when the concentration was up to 400 µg/mL, indicating that isolated peptide (P2) from the EBN by-products could be candidate compounds for antioxidant food and drugs.

In addition to ROS, RNS such as nitric oxide (NO) and peroxide, can also exert deleterious effects on cells [44]. As a NO donor, SNP leads to cellular oxidative stress damage and apoptosis by producing excess NO [45,46]. Therefore, the protective effect of the purified peptides against SNP-induced oxidative damage of PC12 cells was investigated in this paper. As displayed in Figure 8b, the cell viability showed a linear downward trend with the increase of SNP concentration. When the PC12 cells were stimulated with 500 μM SNP for 24 h, the cell viability became 58.96 ± 3.39%, which was close to 60%. Therefore, 500 µM of SNP was selected for the evaluation of peptide oxidative protection activity. As shown in Figure 8c, PC12 cells were pretreated with purified peptides (50~400 μg/mL) for 2 h, cell viability was prominently boosted with a dose-effect (*p* < 0.05). Moreover, 50 μg/mL of the peptides had a weaker protective effect on PC12 cells from SNP-induced injury, while with the concentration up to 400 μg/mL, the cell viability increased to 85.66 ± 1.40%, which was similar to the effect of 100 μM edaravone (positive control). Our finding suggested that the purified peptides (P2) could reduce SNP-induced PC12 cell damage and enhance cell viability, which agreed with the effect of EBN extract to attenuate H_2_O_2_-induced oxidative damage on human neuroblastoma cells [47].

### 3.9. Identification of Antioxidant Peptides by LC-MS/MS

Peptide sequence for the P2 fraction was identified by LC-MS/MS since it had the highest antioxidant activity. As displayed in Table 5, the sequence length of 12 peptides was between 8 and 17 and the molecular weight was about 1000–2000 KDa. The characterized peptides in the P2 fraction originated from Actin, Heterogeneous nuclear ribonucleoprotein, MYH11 protein, Tropomyosin alpha-1 chain, and so on.

Studies have demonstrated that molecular weight, amino acid sequences, compositions, and hydrophobicity of peptides were intimately correlated with their antioxidant ability [48,49]. In this study, the identified peptides were usually composed of 2–20 amino acids with molecular weights between 200 and 3000 KDa, which were similar to the characteristics of typical antioxidant peptides [4]. Moreover, most of the peptides in this study were rich in Arg at the *C*-terminus, the Arg was beneficial to enhance the antioxidant effect of peptides [37]. It has been reported that the lysine residue contained in the *C*-terminus of the peptides was beneficial for improving the radical scavenging ability [50]. For example, hydrophobic amino acids including Try, Met, Pro, Val, Phe, Leu, and Ala may enhance the radical scavenging ability of peptides [51,52]. In this study, one or more hydrophobic amino acids were included in the identified peptides. Furthermore, some acidic amino acids such as Asp and Glu are also regarded as to be vital ingredients of the antioxidant activity of peptides [53]. Therefore, the individual peptides identified in this study may have higher antioxidant activity, and the next step should be to synthesize individual peptides for activity and structure–activity studies.

## 4. Conclusions

In this paper, a response surface methodology was applied to optimize the extraction process of antioxidant peptides from EBN by-products. The EBN by-products protein hydrolysate was further purified through ultrafiltration, G-25 gel chromatography, and RP-HPLC. Finally, 12 peptide sequences were identified by LC-MS/MS. Purified peptides exhibited good antioxidant activities, which effectively scavenged DPPH radical, ABTS radical, and O_2_^−^ radical, and had a protective effect against the oxidative stress damage of PC12 cells induced by SNP. These results suggested that EBN by-products may be a new source of antioxidants. In addition, producing antioxidant peptides from EBN by-products can improve the economic value of EBN by-products. However, the biological activities and structure–activity relationships of synthetic monomeric peptides need to be determined in further studies.

## Figures and Tables

**Table 1:** Optimal reaction conditions of proteases.

Types of Proteases	Optimum Temperature (°C)	Optimum pH	Buffer
papain	65	7.0	hydrochloric acid solution
acid protease	45	3.0	hydrochloric acid solution
neutrase	40	7.0	hydrochloric acid solution
alcalase	55	9.5	sodium hydroxide solution
pepsin	37	2.0	hydrochloric acid solution
trypsin	37	8.0	hydrochloric acid solution

**Table 2:** Levels and factors of the response surface.

Levels		Factors	
	A Enzyme Concentration (kU/g)	B Hydrolysis Temperature (°C)	C Hydrolysis Time (h)
−1	8	40	8
0	−10	50	10
1	−12	60	12

**Table 3:** Design proposal and experiment results of response surface.

Numbers	A: Enzyme Concentration (kU/g)	B: Hydrolysis Temperature (°C)	C: Hydrolysis Time (h)	Y: DH (%)
1	1	−1	0	15.56
2	0	0	0	16.68
3	−1	−1	0	15.86
4	0	1	−1	11.46
5	1	0	−1	16.90
6	0	1	1	11.45
7	1	0	1	15.70
8	0	−1	−1	15.56
9	0	0	0	17.15
10	0	−1	1	16.99
11	−1	0	−1	15.11
12	−1	0	1	15.29
13	−1	1	0	10.60
14	1	1	0	12.36
15	0	0	0	17.06
16	0	0	0	17.35
17	0	0	0	17.15

Notes: DH indicated the degree of hydrolysis, and the data indicated as means ± SD (*n* = 3).

**Table 4:** Regression model variance analysis results of response surface.

Source	Sum of Squares	DF	Mean Square	*F* Value	*p*-Value
Model	80.98	9	9.00	51.21	<0.0001 **
A	1.67	1	1.67	9.53	0.0176 *
B	40.95	1	40.95	233.10	<0.0001 **
C	0.02	1	0.02	0.11	0.7457
AB	1.06	1	1.06	6.04	0.0436 *
AC	0.48	1	0.48	2.71	0.1437
BC	0.52	1	0.52	2.95	0.1295
A^2^	2.69	1	2.69	15.30	0.0058 **
B^2^	30.33	1	30.33	172.65	<0.0001 **
C^2^	1.18	1	1.18	6.71	0.0360 *
Residual	1.23	7	0.18		
Lack of fit	0.99	3	0.33	5.41	0.0682
Pure error	0.24	4	0.061		
Cor total	82.21	16			

*R*^2^ = 0.9850; *R*^2^_adj_ = 0.9658. * *p* < 0.05; ** *p* < 0.01.

**Table 5:** The peptide sequences of the P2 fraction.

Protein Source ^a^	Number	Sequence	Length	Mass (Da)
Actin, cytoplasmic type 5	1	SYELPDGQVITIGNER	16	1961.16
Heterogeneous nuclear ribonucleoprotein A1	2	IFVGGIKEDTEEHHLR	16	2050.31
	3	KIFVGGIKEDTEEHHLR	17	2196.50
MYH11 protein	4	VIQYLAVVASSHK	13	1630.84
Tropomyosin alpha-1 chain	5	HIAEEADRKYEEVAR	15	1954.13
MYH9 protein	6	LKNKHEAMITDLEER	15	1782.94
78 kDa glucose-regulated protein	7	TFAPEEISAMVLTK	14	1890.01
SDK2 protein	8	ATVVTVRP	8	968.11
Spermatid perinuclear RNA-binding protein	9	GLKYELISETGGSHDKR	17	2030.36
RO31 protein	10	AVSREDSVKPGAHLTVK	17	1982.27
Paired amphipathic helix protein	11	EEEEEEEMDVDETT	14	1747.75
CHMP6 protein	12	ALLLLKKKR	9	1226.54

^a^ The protein sources of the identified peptides are from the Uniport protein database.

## Data Availability

Processed data and analysis scripts are available on https://www.iprox.cn/ (accessed on 14 February 2022) ID: IPX0004212001.
